# Canine Detection of the Volatilome: A Review of Implications for Pathogen and Disease Detection

**DOI:** 10.3389/fvets.2016.00047

**Published:** 2016-06-24

**Authors:** Craig Angle, Lowell Paul Waggoner, Arny Ferrando, Pamela Haney, Thomas Passler

**Affiliations:** ^1^Canine Performance Sciences Program, College of Veterinary Medicine, Auburn University, Auburn, AL, USA; ^2^Center for Translational Research in Aging and Longevity, University of Arkansas for Medical Sciences, Little Rock, AR, USA; ^3^Department of Clinical Sciences, College of Veterinary Medicine, Auburn University, Auburn, AL, USA

**Keywords:** volatilome, volatile organic compound, canine detection, disease detection

## Abstract

The volatilome is the entire set of volatile organic compounds (VOC) produced by an organism. The accumulation of VOC inside and outside of the body reflects the unique metabolic state of an organism. Scientists are developing technologies to non-invasively detect VOC for the purposes of medical diagnosis, therapeutic monitoring, disease outbreak containment, and disease prevention. Detection dogs are proven to be a valuable real-time mobile detection technology for the detection of VOC related to explosives, narcotics, humans, and many other targets of interests. Little is known about what dogs are detecting when searching for biological targets. It is important to understand where biological VOC originates and how dogs might be able to detect biological targets. This review paper discusses the recent scientific literature involving VOC analysis and postulates potential biological targets for canine detection. Dogs have shown their ability to detect pathogen and disease-specific VOC. Future research will determine if dogs can be employed operationally in hospitals, on borders, in underserved areas, on farms, and in other operational environments to give real-time feedback on the presence of a biological target.

## Introduction

All living things are susceptible to pathogens and diseases. Scientists have been investigating technologies that aid in early detection, therapeutic monitoring, and prevention of transmission of pathogens and diseases. Recent developments in the field of volatilomics have resulted in novel, emerging technologies that identify pathogens or disease states using characteristic chemical vapor emission patterns.

Dogs are a mobile real-time detection technology that identify targeted chemical vapor profiles. Dogs have been used in operational environments for years as real-time chemical detectors of explosives, narcotics, accelerants, people, animals, and other targets of interest. Dogs possess an extremely sensitive sense of smell, with a demonstrated lower limit of detection at concentrations of one part per trillion (ppt) (1), which is three orders of magnitude more sensitive than today’s available instruments, which can reliably identify substances at concentrations as low as parts per million (ppm) or billion (ppb). To illustrate the tremendous canine olfactory sensitivity, a dog could detect the equivalent of one drop of a liquid in 20 Olympic-size (2500 ft^3^) swimming pools. Their powerful sensory system allows dogs to detect diseases, pathogens, cadavers, lost/criminal persons, and other biological targets. However, much more research needs to be conducted to understand what exactly the dogs are detecting.

Advancements in analytical chemistry in recent years have made it possible to quantify and compare volatile organic compounds (VOC) of cellular origin. VOC are low molecular weight compounds that easily evaporate at normal temperatures and pressure (2). All odorants are VOC, and advancements in VOC analysis have provided a foundation to begin to understand what dogs are detecting in regard to biological targets. Current VOC analysis requires sophisticated stationary analytical chemical instrumentation, such as liquid/gas chromatography–mass spectrometry. These instruments are valuable for the discovery of biological VOC and to identify specific VOC, but unlike dogs, instruments are typically confined to a laboratory, unable to detect VOC in real time, and do not have the capability to track the odor to its source. Diagnostic testing using laboratory instrumentation in an operational environment (e.g., mass transit, cargo, agriculture) is often impeded by the lack of cleanliness, interference by air particulates, presence of non-target VOC produced by various substances in the environment, and the constantly changing variables, such as temperature, humidity, wind, and thermal plumes. Rapid advancements will increase the analytical sensitivity of available instrumentation; however, current instruments are only capable of accurately analyzing compounds in the ppb range (3). In operational environments, there is a need for real-time detection of static and dynamic targets. Analytical instruments cannot fill this capability gap without significant improvements in portability, sensitivity, user knowledge, and the ability to trace an odor to its source.

Dogs provide many force-multiplying advantages in operational environments. Dogs can scan large areas efficiently, which is important for detecting pathogens in large herds of animals, in crowds of people, on or in objects (e.g., ships, airplanes, buildings), or around areas of land. Therefore, from an operational perspective, dogs are a superior technology offering a highly sensitive, real-time sensory system on an intelligent mobile platform without the need to collect, process, or analyze samples, which gives them significant advantages over machines in operational environments. This review paper will discuss cited literature and postulate potential targets of canine detection. Furthermore, we hope to stimulate thought about future advances and discoveries in canine detection of pathogens and diseases. The scope of this review is not to discuss the identity of specific VOC, but to discuss the concepts of VOC identification related to canine detection. Other research and review articles such as by de Lacy Costello et al. (4) have described specific VOC found in diseases and pathogens.

## The Volatilome

Physiological processes of all organisms produce metabolic products, including VOC (5). Production of cellular VOC occurs in millions of cells simultaneously, thus potentially releasing extracellular VOC on a detectable scale. These VOC enter the blood stream and are then released into the air around a human, animal, or plant. The mechanism of the release is through breath, urine, feces, skin emanations, and blood (6). The volatilome is the entire set of VOC produced by an organism. The volatilome is the accumulation of VOC in an organism, and the VOC reflect its unique metabolic state. Animals, humans, insects, and even plants can detect VOC that are released from an organism. For example, Bicchi and Maffei (7) stated that the plant volatilome is involved in critical processes, including plant–plant interactions, the signaling between symbiotic organisms, the attraction of pollinating insects, and a range of biological activities in mammals. Recent research demonstrated that the volatilome can be used to detect disease, pathogens, and many other unique aspects of an organism. VOC are released in concentrations of ppb to ppt in human breath, and ppm to ppb in human blood and urine (3). Dogs were demonstrated to detect *n*-amyl acetate in the ppt range (8), indicating that most of the VOC in the volatilome are within a dog’s capabilities of detection.

Volatile organic compounds are emitted constantly from the human body and consist of hundreds of VOC secreted from cells as a result of metabolic processes (9). Amman et al. (10) stated that more than 1000 VOC appear in exhaled breath, skin emanations, urine, saliva, or feces. de Lacy Costello et al. (4) reported that VOC were identified and assigned in breath (872), saliva (359), blood (154), milk (256), skin secretions (532), urine (279), and feces (381) in apparently healthy individuals. Besa et al. (11) stated that VOC may be products of various inflammatory and metabolic processes, either physiological or disease related, that take place in the airways and other parts of the human body, or products of the oxidative stress that occur in disease states. Variations in these compounds between individuals are great and concentrations may depend on several factors, such as metabolism, differences in lung, or systemic physiologies (11). Schmidt and Podmore (3) stated that VOC patterns differ between individuals because of uncontrolled variables, such as genetic differences, environmental settings, diet, drug ingestion, and smoking. There are many different physiological processes that influence the detectable VOC profile. However, not all VOC released from the body are related to human metabolism. Many VOC are related to the commensal microbiota or from microbial infections. For example, bacteria present in the mouth, lungs, and digestive tract are potent producers of VOC (12). Other VOC may be from foods, drinks, medications, personal hygiene products, and pollutants that are ingested, inhaled, or absorbed through the skin. Thus, the volatilome is a complex combination of endogenous and exogenous VOC, and care should be taken not to label exogenous compounds as disease biomarkers.

The source of endogenous VOC that are part of the volatilome are diverse metabolic processes occurring simultaneously in the various cell types that shape an organism. It is estimated that the average human body contains 37.2 trillion cells (13) that simultaneously conduct metabolic processes, releasing specific VOC unique to the particular type of cell and cellular process. Differences in VOC expression patterns may result from genetic variation among individuals. Aksenov et al. (5) demonstrated that human leukocyte antigen (HLA) alleles can directly influence production of specific volatile compounds at the cellular level. The authors stated that the resulting odor fingerprint depends on the expression of specific HLA sequences, and as a result, a unique VOC pattern or “odorprint” is formed. This specific odorprint is the result of downstream intracellular metabolic pathways that have been influenced by a particular allele. This highly specific and unique VOC profile, derived by metabolic changes at the cellular level, may be useful for imprinting and reliable detection by a properly trained canine. More research is needed to determine if a canine’s olfactory capability can accurately distinguish between complicated VOC profiles of similar diseases, despite the likelihood of many overlapping compounds.

Following intracellular production of the VOC, the VOC may transverse the cellular wall and enter into the blood stream. The blood stream represents the main means of communication with different parts of the body and as such collects information on the metabolic, nutritional, and immunological status of an organism (9). The secreted VOC are transported with the blood to various organs where they are off-loaded. For example, when the blood travels to the lungs, some of the VOC are removed and exhaled in the breath. Other VOC are removed in the kidneys and released in the urine, while VOC released in the feces are a direct reflection of the endpoint of many excretory and secretory processes in the organism (9). Some VOC are secreted through the skin *via* the sweat glands and released from the gut. These VOC either enter the environment directly or are altered by the microbiome and released as altered VOC. de Lacy Costello et al. (4) stated that skin is not homogeneous and the distribution of the different types of glands and bacterial flora across the body can be expected to lead to different VOC profiles. Furthermore, the odors of a single individual vary over time though diet, emotional state, menstrual cycle, age, and many others factors. Therefore, once VOC are produced by cells, they enter the blood stream or intestines and take multiple paths out of the body and into the environment. Once in the environment, the VOC surround the body and may be detected as a unique individual volatilome that allows dogs to detect the biological target. Currently, the field of volatilomics is in its infancy, and the biochemical mechanisms behind the release of disease-specific VOC is largely unknown (3, 9, 14). It is not known if unrelated diseases can produce identical VOC profiles. If disease A and disease B result in identical metabolic changes to a cell, then identical VOC profiles may be released. In this case, false-positive indication by a trained dog would reduce the diagnostic specificity and hamper operational usefulness. Therefore, canine detection research must use disease distractors that cause similar metabolic changes in infected cells (15).

## Biomedical Detection

Recent research interest revolved around the ability of dogs to detect cancer in real time. Working dogs have been successfully used in the detection of other biomedical targets, such as hypoglycemic episodes in diabetic people. Petry et al. (16) stated that glucose-detecting dogs are potentially effective in the detection of diabetes in children. Studies demonstrated that tissues or systems infected with pathogens or affected by disease release unique VOC, which become part of detectable odor signatures (9, 14). For example, Jezierski et al. (17) stated that aberrant protein synthesis and changed metabolisms in cancer cells produce VOC that are likely to have distinctive odors detectable by highly sensitive analytical devices.

### Unique Pathogenic and Disease State VOC

Multiple studies demonstrated that VOC patterns may be specific to diseases or pathogen-specific infection. Abd El Qader et al. (18) demonstrated that metabolic changes in viral and bacterial microbiological cultures are associated with significant VOC released by the pathogens, providing sample fingerprints for both its identity and existence. Schmidt and Podmore (3) stated that different patterns of VOC expression are associated with diseases, such as cancer, asthma, cystic fibrosis, diabetes, tuberculosis, chronic obstructive pulmonary disease, heart allograph rejection, and irritable bowel syndrome. The authors further described that these VOC expression patterns may be caused by pathological processes that generate new VOC not produced during normal physiological processes and/or altered VOC concentrations. Therefore, the production of new VOC or alteration of the VOC expression pattern may serve as a biomarker for the assessment or detection of disease by dogs.

In a review of 31 publications, Bos et al. (19) concluded that many pathogenic bacteria have distinct metabolisms that produce species-specific VOC and suggested that the presence of these VOC in patients indicated infection. In a cell culture model, Schivo et al. (20) demonstrated different VOC expression patterns in primary human tracheobronchial cells infected or uninfected with human rhinovirus. Aksenov et al. (21) determined that VOC produced by B lymphoblastoid cells following infection with three live influenza virus subtypes were unique for each virus subtype. In addition, Abd El Qader et al. (18) examined the VOC released from cultures of five viruses (influenza A, influenza B, adenovirus, respiratory syncitial virus, and parainfluenza 1 virus) and four bacteria (*Moraxella catarrhalis, Haemophilus influenza, Legionella pneumophila*, and *Mycoplasma pneumoniae*). The researchers detected 12 and 6 VOC that were associated with bacterial and viral growth, respectively, and identified 2 VOC that differentiated bacterial and viral infection (18). Lastly, Mashir et al. (22) administered live attenuated H1N1 vaccine (FluMist^®^) to humans and demonstrated that, in exhaled breath, VOC increased for 7 days after the vaccination. If pathological processes, such as infections, neoplasia, and metabolic disorders, influence the type, ratio, and strength of VOC emitted from an organism, then unique VOC patterns may create a specific signature odor (23) for the dog to detect.

### Dogs as a Pathogen and Disease Sensor

Detection dogs are able to detect cancer by sampling breath, feces, urine, blood, and tissue. In some cases, dogs were able to detect disease states in exhaled breath, which contains the lowest known VOC concentrations of the volatilome (3). Sonoda et al. (24) trained a dog to examine human patients with colon cancer using samples of exhaled breath and watery stool. This dog’s sensitivity and specificity for cancer detection in breath samples was 0.91 and 0.99, respectively. The sensitivity and specificity for detection in stool samples was 0.97 and 0.99, respectively. Another study demonstrated that the overall sensitivity of canine scent detection of lung cancer utilizing exhaled breath samples was 0.99, with a specificity of 0.99 (25). In the same report utilizing trained dogs to evaluate breath samples from breast cancer patients and controls, the sensitivity of detection was 0.88 and specificity was 0.98 (25). In addition, Angle et al. (15) demonstrated that dogs could detect bovine viral diarrhea virus and discriminate it from bovine herpesvirus 1 and bovine parainfluenza virus 3, in cell cultures. The diagnostic sensitivity and specificity in this study was 85 and 96% and 98 and 99% for each of the two dogs, respectively. These reports suggest that VOC or similar compounds from diseased internal tissues and cell cultures are released externally and may be detected in the volatilome with trained dogs with a high degree of diagnostic accuracy.

Purpose-bred detection dogs have a demonstrated ability to search for unique odor patterns and identify specific targets in field conditions amidst substantial “odor noise” (i.e., varied and/or strong odors). Although at least 381 unique VOC are emitted from human feces (6), a trained dog was able to detect *Clostridium difficile* in human stool samples (26). The dog detected *C. difficile* with high diagnostic sensitivity and specificity in stool samples and hospitalized patients, correctly identifying 25 of the 30 *C. difficile* cases and 265 of 270 control cases (26). This study emphasizes a dog’s ability to detect a specific odor pattern among the myriad of odors from other bacteria, fungi, and viruses, naturally present in feces, and emphasis its ability to function operationally, as the dog was working in a hospital room and successfully found *C. difficile*. In another article, Alasaad et al. (27) trained two detector dogs to follow the scent of sarcoptic-infected live animals and to find carcasses in the Italian Alps. The authors concluded that properly trained disease-detector dogs are an efficient and straightforward tool for surveillance and control of sarcoptic mange in affected wild animal populations. These two studies show that dogs have the capabilities to detect disease targets in an operational setting among environmental odor noise (i.e., not in an odor sterile laboratory environment).

Most of the studies mentioned in this section only used one or two dogs in simple target distractor arrangements. Moser and McCulloch (28) reviewed the methods and accuracy of studies of canine scent detection of human cancers. The authors noted that variability and inadequacies in reported training methods, target and control sample preparation and presentation, utilized dog numbers, and a paucity of peer-reviewed articles made it difficult to assess the general capability of trained dogs as a diagnostic tool for the detection of cancer. Further research with more dogs and more complex targets and distractor arrangements is crucial to determine the feasibility of dogs as operational biological detectors.

### Disease Detection by Free-ranging Predators

From a comparative biology standpoint, there may be much that can be learned from the detection dog model about the behavior of free-ranging predators. With the knowledge that disease and pathogen detection is within the canine olfaction capability and that diseases can be detected outside the body, the question arises in which other ways animals might utilize this extraordinary sensory capability in the wild. Do animals learn to hunt prey that is diseased or stressed based on VOC biomarkers? Animals that hunt herd animals often stalk their prey and select slow and diseased animals, prompting the question: are they using their olfaction capabilities to find potentially diseased animals? This is not a farfetched idea as the dogs cited in the research papers, in this review, were rewarded with a toy or food for identifying the target, in the wild they are rewarded with the satisfaction of a meal. Considering an animal might feed daily or weekly, there is considerable opportunity to learn patterns (olfaction, auditory, and vision) of successful kills. Therefore, it is possible that animals learn with experience from the prey that they kill and that there are particular odors associated with animals they can catch and do not catch. Future research utilizing detection dog models could give a better understanding of how animals might use their sense of smell to identify the volatilome of weak and diseased animals for hunting.

## VOC Movement Outside the Body

There is no published literature on the movement of the odorous VOC. The human thermal plume and human aerodynamic wake may serve as a model for the movement of the detectable odorous volatilome. Craven and Settles (29) stated that knowledge of the behavior and underlying physics of the human thermal plume is essential to the study of contaminant transport from people and the understanding of the entrainment of respirable particles into the human breathing zone. It is important to determine if the detectable odorous volatilome moves around the body and away from the body, as future discoveries may show that dogs could sense biological VOC better in certain regions around the body for the purposes of non-invasive sampling.

When a human body and an environment differ in temperature, a temperature gradient forms in the surrounding air that drives buoyant convection about the human. Experiments and computational fluid dynamics simulations demonstrated that the maximum plume velocity is about 0.2–0.3 m/s and plume flow rates are in the 20–80 L/s range, depending on activity levels, height above the human subject, and characteristics of the indoor environment (30). Entrapped in these convective currents are VOC, pathogens, and even skin cells. For example, Craven and Settles (29) described that exfoliated human skin is the most prevalent and transported particulate in the thermal plume. On average, a complete layer of skin is shed every 1–2 days, releasing a million skin cells per minute. Most inhaled air comes from the human boundary layer that contains these particles, from which 6000 to 50,000 particles/L of air enter the human nose. Jia et al. (31) demonstrated that the thermal plume was capable of dispersing solid particles (5 μm in diameter) deposited on the floor by the legs. The particles traveled up the body, over the head, and into the area above the head toward the ceiling of a small room. Therefore, transportation of VOC by the thermal plume, which moves VOC around and away from the body, is plausible and likely. This hypothesized movement of VOC should be considered and verified, as it could lead to concentrated sampling positions for non-invasive canine detection.

Craven and Settles (29) described the human thermal plume as a potential whole-body chemical trace sampling system. “If the plume is collected and sampled, its contaminant burden may be analyzed for various purposes, including medical diagnosis and substance detection.” That study referred to the thermal plume of static subjects in a sitting position and not dynamic subjects walking about. Walking subjects change the aerodynamics of the volatilome and, ultimately, the search technique and sampling positions of the dog. As a person walks, air streams behind them in their wake. Craven et al. (30) described that the overall effect of free convection about the body is diminished and the human thermal plume bends over at an angle with respect to the flow direction. As free-stream velocity increases (as a person walks faster), the flow about the body becomes dominated by horizontal forced convection and the temperature difference between the body and the air ceases to be relevant. At this point, the term “human thermal plume” is dropped and the flow now is called the “human aerodynamic wake.” The authors described the human aerodynamic wake as the unsteady airflow downstream of a walking person (or a motionless person standing in a breeze). This is an important concept, as, in theory, the volatilome may stream behind the body in this wake and be available for detection by the dog. More research into static and dynamic subjects needs to be conducted in order to understand VOC concentration zones within the volatilome for the purposes of identifying specific canine search patterns, which target optimal VOC zones for non-invasive detection.

Dogs search the volatilome in two main ways, as static objects in containers (e.g., urine sample head space) in a laboratory environment or by actively searching for a target in an operational environment (e.g., trailing a human subject). It is important to understand the movement of the detectable odorous volatilome in order to maximize a dog’s capability to detect the desired target. The fluid dynamics of the human thermal plume and aerodynamic wake of both organisms and inanimate objects in conjunction with wind currents dictate optimal micro- and macro-search patterns for canine detection. Micro air currents occur immediately around the target, while macro currents occur downstream from the target. The dog uses odor pools and air currents (which contain odor) to follow target odor back to its source. The air currents in conjunction with the geometry and surface texture of surrounding objects influence the behavioral characteristics of the target odor. The behavioral characteristics of the target odor directly influence the search pattern of the dog and, ultimately, the training of the dog to enhance its macro- and micro-search patterns in the target odor zone. Much more research needs to be conducted in order to understand the movement of biological VOC within the thermal plume (e.g., micro currents) and in the aerodynamic wake/wind currents in order to develop search patterns to optimize biomedical detection.

## Conclusion

Dogs and analytical chemical instrumentation have verified that pathogens and diseases produce unique VOC signals, which can be detected outside the body. There are many uses for dogs in the detection of pathogens and diseases because of their outstanding mobility and sensory capacity. Increasing knowledge about the detectable odorous volatilome will aid to train dogs to detect specific biomedical targets. By uncovering volatilome characteristics and behaviors, productive search areas and targets will be identified. This will help increase the portfolio of operational uses for detection dogs. Conversely, studying the VOC signatures that control the detection responses of dogs to particular pathogens may inform the design of detection instrumentation.

There are many potential operational uses for detection dogs to detect biological targets. Further understanding of the volatilome will lead to discoveries of how to utilize the dog’s highly sensitive detection capabilities. Below is a list of potential uses of detection dogs and areas that need further research:

- Dogs could be used in fields such as analytical chemistry to identify trace materials in compounds that currently cannot be quantified due to a lack of instrument sensitivity. This could lead to new discoveries and challenges in chemical analysis.
- Dogs could be utilized as a model for sensor development for mobile real-time sensors in operational environments.
- Dogs could be used in underserved areas to identify crop, livestock, and human diseases. These applications would enhance food security, biosecurity, and prevention and containment of bio- and agro-terrorism.
- Dogs could be used in hospitals to identify pathogens and diseases (e.g., methicillin-resistant *Staphylococcus aureus*) that could spread throughout the hospital.
- Dogs could be used as point-of-care diagnostics for plants, humans, and animals to detect pathogens and diseases that currently do not have an available real-time diagnostic test.
- Dogs could be used to establish disease outbreak containment zones where they could screen “things” (i.e., people, vehicles, items) coming out of the containment zone.

The dog’s sensory system affords it the ability to detect biological targets. The biological targets are very complex and their detection is intricate. There are challenges faced by the canine industry because of the wide range of performance capabilities by the dogs in biological detection. One of the main challenges is how to employ the appropriately trained canines for the task. Researchers should use purpose-bred dogs for detection and select dogs with highly sensitive olfactory capabilities, innate search techniques optimal for biological detection, and calculated methodical skill sets. Researchers should utilize highly skilled professional trainers with an in-depth understanding of detection dog behaviors. There are intricacies in training dogs on biological targets that are complex and unlike any skill set utilized in other aspects of dog training. A detection dog is a highly complex sensory technology, and not understanding its full capabilities and its influences could skew study results to not reflect the true potential of a dog. Lastly, researchers should carefully consider biological target research methodologies to reduce specificity issues and the inflation of sensitivities that are not related to the target odor. Future research should address the above issues.

Studies demonstrated that the VOC present in the volatilome are well within the detection capability of the dog. Dogs have shown their ability to detect pathogen and disease-specific VOC. Future research will determine if dogs can be employed operationally in hospitals, on borders, in underserved areas, and in counter-terrorism operations to give real-time feedback on the presence of biological targets.

## Author Contributions

CA, LW, AF, PH, and TP developed the literature review idea and need for this knowledge within the industry. All authors co-wrote the manuscript, and read and approved the final manuscript.

## Conflict of Interest Statement

The authors declare that they have no conflicts of interest. There are no MTAs, patents, or patent applications that apply to reagents, methods, or data in the review paper.
